# Cardiotoxicity in people undergoing cancer treatment - the role of the oncology nurse

**DOI:** 10.1016/j.apjon.2024.100597

**Published:** 2024-09-30

**Authors:** Geraldine A. Lee, Priya Reehal

**Affiliations:** a Professor of Nursing and Chair of Health Service Research, Catherine McAuley School of Nursing & Midwifery, Brookfield Health Sciences Complex, University College Cork, Cork, Ireland; b Cardio-Oncology/Heart Failure Clinical Nurse Specialist, Cardio-Oncology Centre of Excellence, Royal Brompton Hospital, Part of Guy's and St Thomas' NHS Foundation, London, United Kingdom

**Keywords:** Cardiotoxicity, Oncology nurse, Cardiovascular assessment, Cardio-oncology

## Abstract

Cardiotoxicity is a significant complication of cancer treatment, and this review describes the cardiovascular risks associated with various therapies and emphasizes the crucial role of oncology nurses in managing these risks. Many cancer treatments, including cytotoxic therapies, radiation, targeted therapies, and immune-modulatory drugs, have been shown to increase the likelihood of cardiovascular toxicity, leading to conditions such as acute coronary syndrome, heart failure, and atrial fibrillation. Guidelines are now available to reduce treatment-related cardiovascular toxicity (CTR-CVT) and stress the importance of cardiovascular assessments before, during, and after cancer treatment. Oncology nurses are pivotal in performing these assessments and collaborating within multidisciplinary teams, including cardiologists, to ensure optimal care. As more patients successfully complete cancer treatment, it becomes essential to integrate cardiovascular risk evaluation, education, and medication into routine oncology care. The evolving field of oncology nursing, particularly with the rise of emerging therapies and aging populations, requires further education on early detection and management of cardiotoxicity to enhance patient outcomes.

## Introduction

People are now living longer and during their lifetime may have a cancer diagnosis and have cardiovascular disease. For those undergoing cancer treatment, a significant proportion will have a pre-existing cardiovascular condition (such as hypertension or raised cholesterol) while others will be at risk of having a cardiovascular event such as an acute coronary syndrome or heart failure as a result of their cancer treatment. With a cancer diagnosis and an underlying cardiovascular condition, patients often require specific input from these two distinct specialties and as a result of the demands on these services, this has led to the establishment of cardio-oncology as a specialty. The goal of cardio-oncology is the treatment of cardiovascular disease (CVD) in those who are going or have undergone cancer treatment as there is an increased risk of cardiotoxicity from cancer therapies. The first European Society of Cardiology guidelines were published in 2022 and they highlight the complexity of care and the need for better co-ordination between the two specialties.^1^ For health care professionals (HCPs) working in cardio-oncology, there is a need to gain knowledge and skills from oncology and cardiology. However, there are gaps in this emerging field and surveys undertaken among those specialising in cardiology highlighted the need for close collaboration with oncology services.^2^^,^^3^ The surveys also highlighted the need for cardio-oncology education for all HCPs and the importance of a multi-disciplinary approach to patient care as well as more information about cardiotoxicity in terms of assessment and management.^2^^,^^3^ There is growing evidence that long-term cancer survivors are more likely to die from CVD than the rest of the population and therefore, risk stratification of patients for CVD as well as management and surveillance of cancer patients and cancer survivors who have or develop CVD is critical if we are to improve outcomes.^4^^,^^5^ Specifically from a nursing perspective, nurses working in cardio-oncology need to have the appropriate skills and knowledge for both specialties and have more than likely worked in cardiology or oncology before their move into cardio-oncology, especially in their role of assessing for and managing cardio-toxicity during and following cancer treatment.^6^ Therefore, the aim of this paper is to outline the cardiotoxic effects of cancer therapies and describe the role of the oncology nurse in performing cardiovascular assessment in patients with cancer and describe how a risk stratifying assessment tool can be applied to their clinical practice using the evidence from the literature.

An overview of the literature was conducted to assess the cardiotoxic effects of cancer therapies and their associated cardiovascular complications. Relevant guidelines were reviewed to present an evidence-based perspective, identify knowledge gaps, and provide a synthesis of the available evidence. This approach aimed to offer insights that could inform clinical practice and highlight areas where further research and education are needed in oncology nursing.^7^

## Cancer and cardiovascular disease

Cancer and CVD have common risk factors including ageing, smoking and obesity. There is now growing evidence that modifiable ‘lifestyle’ related risk factors contribute to about 30% of cancers.^8^ As well as smoking and obesity, other modifiable risk factors include poor diet (high in saturated fats and processed foods and low levels of fruit and vegetables), excessive alcohol consumption and lack of exercise. Not surprisingly, all these modifiable risk factors are also associated with CVD. Studies have highlighted the negative effects of CVD in those with cancer. One U.S. study noted the importance of traditional risk factors including hypertension, diabetes and dyslipidaemia and higher rates amongst the cancer cohort than those who did not have cancer. Critically, a higher 8-year survival rate was seen in those without CVD (81%) compared to those with CVD (60%).^9^ Not only are there CVD risk factors in cancer patient population but these risk factors increase the risk of acute CV complications including heart failure (HF) and Acute Coronary Syndrome (ACS). These presentations may present during cancer treatment or following completion of treatment. One U.K. study of cancer survivors (*n* = 108,215) recorded increased risks of HF, cardiomyopathy, arrythmia, pericarditis, coronary heart disease, venous thromboembolism and valvular heart disease.^10^ Given the increased risk of CVD deaths in those with cancer, it is critical that health care providers undertake early assessment of cardiovascular risk factors and obtain baseline data on cardiac function. Oncology nurses are well placed to undertake this but require the appropriate education and training. Some countries now have established cardio-oncology centres and this allows a structured and integrated approach to delivery optimal care. However, this is not universal practice. As populations live longer, there will be increasing numbers of cancer survivors with one study predicting 22 million in the U.S. by 2032 with the majority (67%) over 65 years of age.^11^

### Cancer treatment and cardiotoxicities

Cancer treatments continue to evolve with new therapies introduced. However, as survival improves with cancer therapies, there is the added risk of acute and chronic cancer treatment-related cardiovascular toxicity (CTR-CVT). Cytotoxic therapies, radiation, targeted therapies and immune-modulatory drugs are all known to cause acute CV (cardiovascular) events. One registry suggests it occurs in nearly 40% of cancer patients.^12^

Treatment that cause CV complications (acute coronary syndrome, heart failure or atrial fibrillation for example) are known as cardiotoxic therapies and once detected, the cancer therapy needs to be interrupted until the acute CV event has resolved and been treated.^13^^,^^14^ Not only is there a risk of CTR-CVT during treatment but also following completion of treatment.^15^^,^^16^ Therefore close monitoring is necessary during treatment as well as surveillance in cancer survivors following cessation of treatment and the oncology nurse has a key role to play in this.

Cytotoxic drugs can be cardiotoxic and the oncology team need to assess each patient prior to commencing treatment. Anthracyclines, alkylating agents, platinum containing drugs and fluoropyrimidines are associated with CV complications. For patients undergoing radiation therapy, there is a risk of pericarditis either during, or immediately after treatment. In the longer-term radiation can also cause conduction abnormalities, coronary heart disease and valvular disease. Some targeted therapies have also been recognised as being cardiotoxic and these include anti-oestrogens and all-trans retinoic acid) which can cause acute CV presentations. Monoclonal antibodies and small molecule tyrosine kinase inhibitors (for example: gefitinib, erlotinib, sorafenib, sunitinib, and dasatinib) have also been associated with CTR-CVT. Hypertension can be seen in those undergoing therapy with vascular endothelial growth factor (VEGF) therapies and therefore knowledge of which cells are being targeted is useful in determining the possible CV complications. Given the complexity of available treatments for the different cancers, each patient must be assessed on a case-by-case basis using an multidisciplinary team (MDT) approach to assess the benefits and risks of the proposed treatment.^17^^,^^18^

For those with CTR-CVT, a rapid diagnosis is needed so that timely and appropriate treatment can take place. There needs to be an individualized and multidisciplinary approach and the oncology nurse is central to this. Before recommencing the cancer therapy a risk-benefit evaluation needs to be undertaken with the focus on restarting the therapy or changing to an alternative cancer therapy. Some of the acute CV presentations that are reported in patients with cancer are ACS, HF, arrhythmia, venous thrombosis and pericardial diseases and the European Society of Cardiology (ESC) has produced two consensus documents outlining the assessment and management of CTR-CVT complications.^13^^,^^14^

## The nurse's role

For an oncology nurse, as well as preparing the patient for their cancer treatment and providing education on side-effects from the cancer therapies, the nurse will now need to incorporate cardiovascular risk assessment into their practice.^6^ Where there is an established cardio-oncology service that includes a cardio-oncology nurse, this process will be straightforward. However for oncology services that do not have a specialist cardio-oncology nurse, the oncology nurse may be required to undertake a cardiovascular assessment.

Some of the components of the assessment are familiar to all nurses- blood pressure and pulse, along with height and weight that allows body mass index (BMI) to be calculated. However, this is only a small part of the cardiovascular assessment. The patient will also require a 12-lead electrocardiogram (ECG) to detect for underlying arrhythmias (that may be undiagnosed) and a transthoracic echocardiogram to record left ventricular function known as ejection fraction as well as checking the heart valves (for regurgitation and stenosis) along with wall motion in the heart. A thorough medical history needs to be obtained that includes any previous cardiac events, family history of CVD, recording of any modifiable risk factors along with blood tests for lipid profile and other biomarkers.

Regardless of planned cancer therapy, it is advised that all patients undergoing cancer treatment have a baseline CV assessment so that baseline data is obtained. This should include:

1. A comprehensive clinical assessment: an assessment of the cardiovascular system and respiratory system and including resting heart rate, blood pressure, oxygen saturations.
2. Previous medical history (especially if the patient has had a previous history of CVD, especially hypertension and hypercholesterolaemia as well as checking for diabetes and obesity (BMI should be recorded).
3. Modifiable risk factor assessment–physical activity level, diet, smoking, alcohol consumption.
4. Diagnostic tests: lipid profile along with a 12-lead ECG and echocardiogram and biomarkers.

Following history-taking and cardiovascular assessment, the oncology nurse will need to determine what is the patient's risk in relation to their planned cancer treatment. Risk stratification is important and allows early identification of moderate to high risk patients and allows HCPs to have a treatment plan with clear documentation on the recommended monitoring and surveillance.

### Risk stratification

Following this, the clinical assessment findings, along with the medical history with identified risk factors and the results of the diagnostic blood tests need to be reviewed. If the oncology nurse is not confident in reviewing this information, it should be undertaken in conjunction with a cardio-oncologist specialist or a cardiologist (a physician or a nurse). There are assessment tools specifically for oncology patients and the Heart Failure Association (HFA)-Cardio-Oncology cardiovascular risk assessment tool is recommended.^15^ The tool was developed in 2020 and offers a personalised approach to determine baseline risk assessment prior to cardio-toxic anti-cancer therapy. The tool is able to determine risk based on the planned treatment and stratifies risk accordingly into: low risk, moderate risk, high risk or very high risk. Based on the risk, treatment can then be discussed in an MDT meeting where the decision can be made to go ahead with the planned treatment (in the case of low risk) or for those at high and very high risk, a personalised management plan can be devised following a referral to the cardio-oncology or cardiology team. The plan would include medication to lower cardiovascular risk factors such as hypertension and hypercholesterolemia. The MDT is important for specialist input, the aim is to reduce time cancer treatment is waiting to be commenced or is being interrupted. For patients that have a new cardiovascular complication, cancer treatment may still be started or uninterrupted and this is known as ‘permissive cardiotoxicity’. This arises when benefit outweighs the risk; risk being the severity of the cardiac complication. In these instances, the Cardio-Oncology CNS would have the awareness the patient undergoing treatment is high risk and would therefore follow the guidance for monitoring a high-risk patient. The MDT helps to assess and manage the patient to therefore establish their overall risk and benefit. This shows the importance of ensuring all cardiovascular risk factors have been documented so that an accurate risk assessment is in use. Often, the oncology nurse is aware what to monitor; signs and symptoms, together with appropriate tests to include in routine tests, such as cardiac biomarkers; NTproBNP and Troponin I.

The HFA-Cardio-oncology assessment tool has some limitations as it only provides a risk assessment for specific planned treatment. These are anthracycline chemotherapy, human epidermal growth factor receptor 2 (HER-2) targeted therapies, VEGF inhibitors, combination of RAF and MEK inhibitors, multi-targeted kinase inhibitors for CML and multiple myeloma therapies (HFA-ICOS Cardio-Oncology cardiovascular risk assessment tool (cancercalc.com) Table 1.^15^ The website is very useful and offers risk scoring for each therapy such (as seen below). The tool uses several parameters to assessment the risk including previous history of CVD, cardiac biomarkers, age, cardiovascular risk factors and previous cardio-toxic treatment along with the patient's lifestyle related risk factors (smoking and obesity).

**Table 1:** Example of HFA-ICOS Cardio-Oncology Cardiovascular Risk Assessment for anthracycline therapy.

Anthracycline chemotherapy:
**Very high risk factors**
Heart failure or cardiomyopathy
**High risk factors**:
Myocardial infarction or previous coronary revascularisation, stable angina, severe valvular heart disease, baseline LVEF < 50%, age ≥ 80 years, previous anthracycline exposure, prior radiotherapy to the left chest or mediastinum.
**Medium risk factors (2 points each):**
Borderline LVEF of 50%–54%, age 65–79 years.
**Medium risk factors (1 point each):**
Elevated baseline troponin, elevated baseline BNP or NT-proBNP, hypertension, diabetes mellitus, chronic kidney disease, previous non-anthracycline based chemotherapy, current smoker or significant history, obesity.

HFA-ICOS, Heart Failure Association of the European Society of Cardiology and the International Cardio-Oncology Society; LVEF, left ventricular ejection fraction; BNP, brain natriuretic peptide; NT-proBNP, N–terminal pro–B–type natriuretic peptide.

Surveillance is a key part of the assessment tool and there is specific advice about what to do at baseline, during and post completion of treatment. This provides an evidenced-based approach to risk factor management that can be used by the clinical team and explained to the patient and their family. Surveillance includes blood pressure monitoring, 12-lead ECG, serum Troponin and BNP and transthoracic echocardiogram and the oncology nurse may play a key role in co-ordinating these tests and liaising with the patient and their family. It is vital the oncology nurse is aware when these tests should be organised for the patient and ensure patients post treatment are not lost in follow-up by both the cardio-oncology and oncology teams.

The American Heart Association has also published a guide on the US and European guidelines that can be used in clinical practice and includes details on monitoring strategies for those at high risk as well as information on how to manage cardiotoxicity.^17^

### Long term management

**Fig. 1:**
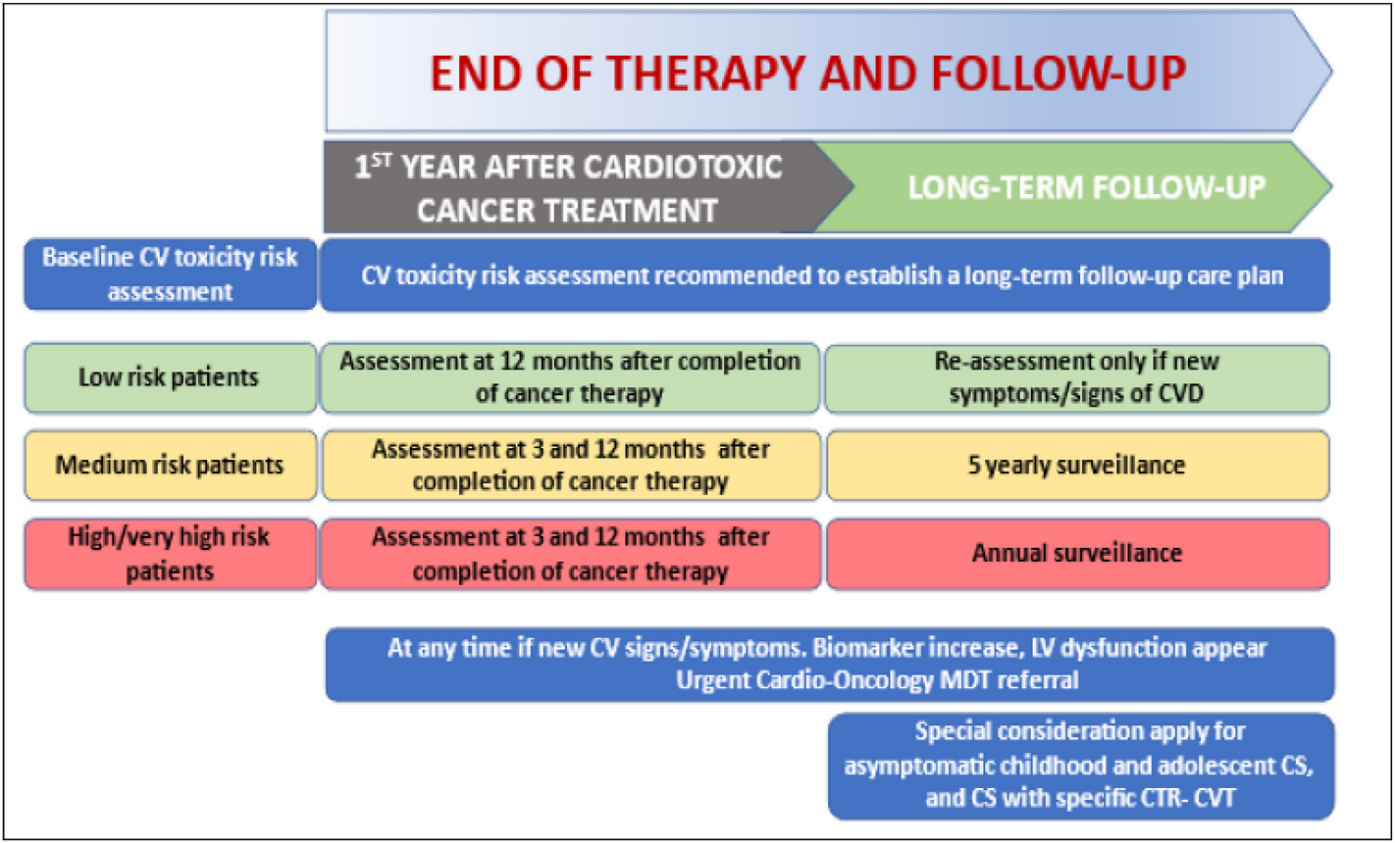
**End of therapy and follow-up from ESC guidelines.** CTR-CVT, cancer treatment-related cardiovascular toxicity; CV, cardiovascular; CVD, cardiovascular disease; CS, cancer survivor; LV, left ventricular; MDT, multidisciplinary team; ESC, European Society of Cardiology.

As previously highlighted, in those with established CVD or at high risk of CRT-CVT at the end of their therapy, periodic evaluation and ongoing surveillance needs to be considered. The ESC 2022 cardio-oncology guidelines (Fig. 1) have outlined the end of therapy and follow-up based on the risk of CV complications and CRT-CVT.^1^ The emphasis is on the timing of risk assessment and surveillance but does not include information on how long-term management of the modifiable risk factors should be approached. As the oncology nurse may have an ongoing therapeutic relationship with their patients, they may be best placed to assess and educate patients about their life-style related risk factors as part of long term follow-up. There is evidence demonstrating that risk factor modification, especially dietary and increased physical activity levels can have a significant effect on reducing the risk of cancer recurrence.^19^ More recently, cardio-oncology rehabilitation has been described and recognises the importance of long term risk factor management and an integrated care approach.^20^^,^^21^ One study of an 8-week rehabilitation programme highlighted the cardiovascular benefits in the intervention group with improved blood pressure, BMI and higher levels of physical activity compared to usual care.^22^ Critically, participants in the intervention group also reported an improved quality of life and health literacy and cost-effectiveness.^22^^,^^23^ It is apparent that long term education and advice on risk factor management is the key to survivorship and in the absence of dedicated cardio-oncology services, the oncology nurse, along with the local cardiology services, could play a central role in this.

### Shared-decision-making

Cancer care needs to be patient-centred with shared decision-making and any discussions about current cancer treatment in the presence of an acute CV event need to be open with the patient and their family fully informed about the situation.^24^

With a conversation around continuing or terminating cancer treatment, an evaluation about the risks and benefits needs to take place with all relevant parties. This may be time- critical but the aim should be to have a MDT discussion with the patient, and family, if applicable, at the centre. The discussion may involve the oncologist, the cardiologist, the haematologist, the radiologist as well as allied professionals including the oncology nurse along with the pharmacist, dietician, physiotherapist and psychologist for example. From a patient's perspective, they may be extremely concerned about any disruption to their cancer treatment and as such will need to be informed about the risks of continuing treatment versus the risks of stopping treatment along with a discussion on what treatment needs to be undertaken for their CV presentation.

### Educating the patient and their family

Given the potential risk of CTR-CVT, patients and their families need to be educated about signs and symptoms and what to do if new symptoms occur. Early diagnosis of CTR-CVT and CV complications are critical and as it may occur when the patent is at home, they need to aware of which HCP and service (i.e., cardiology, oncology or emergency department for instance) to contact. Acute CV complications present with chest pain, shortness of breath or palpitations and these patients should be advised to attend their local emergency department without delay. In addition to being fully informed of the possible CV complications, patients will need to know their stratified risk (i.e., low, moderate or high) as well as the specific signs and symptoms relating to their treatment. Chest pain for example, could be due to an ACS, pericarditis, HF or an arrhythmia. Regardless of the possible cause, they should seek medical advice as they will need a comprehensive cardiovascular assessment so that a diagnosis can be made and treatment initiated. As with any acute cardiac event, early intervention is key, regardless of medical history.

Together with planning follow-up, it is important that patients are aware of the timing of their CV assessment so that they can ensure it occurs at the end of treatment, at one year follow-up and that there is long-term follow-up (if indicated). Patients should be advised about which tests are required (i.e., is it an echo or blood tests or other tests) and whether these will be performed by their primary care team or a dedicated hospital service. This approach ensures that the patient and their family are part of the decision-making and can alert their oncology nurse if they do not receive information about their expected follow-up tests.

One aspect that is often overlooked is how information is shared.^25^ As well as verbally going through the planned monitoring and follow-up, patients should be given written documentation that they can access and share with their family and primary care team.

There is a lay summary document about cardio-oncology treatment and follow-up available from the European Society of Cardiology and oncology nurses could incorporate this into their practice.^26^ However, for the oncology nurse to be able to undertake these additional responsibilities, there needs to be adequate education and training in the field of cardiovascular assessment. To date, there is no dedicated curriculum for nurses with only a core curriculum for physicians is available.^27^^,^^28^ A recent gap analysis has been undertaken amongst nurses and it highlighted the need for greater education by both oncology and cardiology nurses. A core curriculum is now being developed and will be available by 2025 and it will be accessible to nurses online. The aim is to provide nurses with appropriate guidance, support and education in this evolving field.

### Patient experiences

One qualitative study from Australia presented a thematic analysis from 15 interviews and reported the benefits of dedicated cardio-oncology clinics. The themes demonstrated that the cardio-oncology clinic promoted information and understanding, integrated care was evident and managing past and emerging CV risk factors were valued by patients.^29^ However, patients also commented that there were gaps in education and support with the need for more information about lifestyle changes so that they could reduce their risk of CVD. The participants commented on the importance of having clear explanations on their treatment and having their CVD symptoms discussed in a calm and reassuring manner. This highlights the importance of good communication skills and using shared decision-making by the oncology nurse. Quality of life was not included as a quality indicator in a review and clearly more patient-reported outcomes need to be included in cardio-oncology research.^30^ There is a clear need for nurse-led cardio-oncology care where CV risk stratification, management and education is provided with an emphasis on CV risk factors management and lifestyle related advice.^31^

## Conclusions

Oncology nursing is transforming with evolving and emerging therapies and associated risk of cardiotoxicity in an ageing populations with pre-existing CV risk factors or established CVD, especially with the evidenced based guidelines for cardio-oncology. The implications from the current literature strongly support the need for further education and training of oncology nurses on the early detection and risk stratification of cardiotoxicity. With a core curriculum available from 2025, this should lead to increased knowledge of cardiotoxicity in the treatment of cancer amongst oncology nurses and potentially lead to more nurse-led research in the field of cardio-oncology. Possible areas for future research could be on determining if optimal management of pre-existing and acute CV presentations is evident in clinical practice, evaluating current practice and identifying which aspects of oncology care need to be improved.

## CRediT authorship contribution statement

Geraldine Lee- conceptualization, methodology, writing. P. Reehal- editing and writing of draft. All authors had full access to all the data in the study, and the corresponding author had final responsibility for the decision to submit for publication. The corresponding author attests that all listed authors meet authorship criteria and that no others meeting the criteria have been omitted.

## Ethics statement

Not required.

## Funding

No external funding was received.

## Data availability statement

Data availability is not applicable to this article as no new data were created or analyzed in this study.

## Declaration of generative AI and AI-assisted technologies in the writing process

No AI tools/services were used during the preparation of this work.

## Declaration of competing interest

The authors declare no conflict of interest.
